# A mutation in porcine pre-miR-15b alters the biogenesis of MiR-15b\16-1 cluster and strand selection of MiR-15b

**DOI:** 10.1371/journal.pone.0178045

**Published:** 2017-05-24

**Authors:** Wenyang Sun, Jing Lan, Lei Chen, Jinjie Qiu, Zonggang Luo, Mingzhou Li, Jinyong Wang, Jiugang Zhao, Tinghuan Zhang, Xi Long, Jie Chai, Zunqiang Yan, Zongyi Guo, Shuangbao Gun

**Affiliations:** 1 College of Animal Science and Technology, Gansu Agricultural University, Lanzhou, China; 2 Key Laboratory of Pig Industry Sciences (Ministry of Agriculture), Chongqing Academy of Animal Science, Chongqing, China; 3 Department of Animal Science, Southwest University, Rongchang, Chongqing, China; 4 Institute of Animal Genetics and Breeding, College of Animal Science and Technology, Sichuan Agricultural University, Chengdu, Sichuan, China; 5 Gansu Research Center for Swine Production Engineering and Technology, Lanzhou, China; University of Texas MD Anderson Cancer Center, UNITED STATES

## Abstract

MicroRNAs (miRNAs) are small non-coding RNAs that are involved in translational regulation of the messenger RNA molecules. Sequence variations in the genes encoding miRNAs could influence their biogenesis and function. MiR-15b plays an important role in cellular proliferation, apoptosis and the cell cycle. Here, we report the identification of a C58T mutation in porcine pre-miR-15b. Through *in vitro* and *in vivo* experiments, we determined that this mutation blocks the transition from pri-miRNA to pre-miRNA, alters the strand selection between miR-15b-5p and miR-15b-3p, and obstructs biogenesis of the downstream miR-16-1. These results serve to highlight the importance of miRNA mutations and their impacts on miRNA biogenesis.

## Introduction

MicroRNAs (miRNAs) are endogenous non-coding RNAs, ~22 nt in length, that function as negative regulators of gene expression at the post-transcriptional level [1–3], exerting pervasive regulatory impact on nearly all biological processes [2,4,5]. The canonical biogenesis process of miRNAs in animals involves, first, RNA polymerase II-mediated transcription of the genes encoding the miRNAs, resulting in primary transcripts (pri-miRNAs) [6]. In the second step, the pri-miRNAs are then cleaved by the Microprocessor complex of two RNase III enzymes—Drosha and DGCR8—to liberate hairpin-shaped precursor transcripts (pre-miRNAs) [7,8]. In the third step, the pre-miRNAs are exported to the cytoplasm by the protein exportin 5 [9], where, finally, they are cleaved by another RNase III enzyme—Dicer—to release the ~22 nt miRNA duplex [10]. For functionality following the biogenesis process, one strand of the duplex—the ‘guide’ strand—is preferentially loaded into an Argonaute (AGO) protein, forming the RNA-induced silencing complex (RISC), while the free strand—the ‘passenger’ strand—is presumed to be released and degraded quickly [11,12].

The biogenesis of miRNAs is under precise control. Sequence variations in miRNA genes, involving the seed sequence of mature miRNA as well as that of the precursor region, has the potential to affect the precise control of this mechanism by altering the processing of miRNAs; indeed, such variations have been implicated in many human diseases [13–15]. For example, a single nucleotide polymorphism (SNP) in the mature human miR-125a was found to block the processing of pri-miRNA to pre-miRNA, thereby affecting its function of translational suppression [16]; this mutation has since been associated with various human cancers [17,18]. Similarly, mutations in the seed region of human miR-96 were found to interfere with its biogenesis, the detriment manifesting as progressive hearing loss [15]. In addition, SNPs outside of the mature miRNA sequence have also been shown to influence biogenesis and function of the miRNA [19]. For example, a SNP located 24 bp from the 3′ end of mature miR-126 was demonstrated to alter the processing of pri-miR-126 as well as to weaken its suppression function on its target mRNAs [20]. Another example is that a C→T homozygous substitution was found in human pri-miR-16-1, 7 bp in the 3′ direction after the precursor, can cause low levels of miRNA expression *in vitro* and *in vivo* and was associated with prognosis and progression in B-cell chronic lymphocytic leukemia[13]. Moreover, a point mutation in the New Zealand black mice in the flanking region 3′ to the stem loop structure of the pre-mir-16-1 was also found to associated with B-cell chronic lymphocytic leukemia[21].

MiR-15b has been recognized as an important regulator of genes involved in the cell cycle and apoptosis, and has been characterized as dysfunctional in various cancer cell types, [22–24]. The gene encoding miR-15b itself is located in a cluster with miR-16-2. MiR-15b and 16–2 have been shown to modulate the gene expression of various cyclins and growth factors, including *CCND1* and *CCND2* (encoding Cyclin D1 and D2, respectively) as well as *IGF1R* (encoding the insulin-like growth factor 1 receptor); this regulatory mechanism affects proliferation and anti-apoptosis processes, as shown in mouse B cells and in miR-15b/16-2 knockout mice, leading to development of B-cell lymphomas in the latter [25]. MiR-15b/16 were also, more recently, found to contribute to immune mechanisms, enhancing the development and induction of regulatory T cells through their regulation of the hypoxia inducible factor-2α (HIF-2α) [26] and the rapamycin (mTOR) signaling pathway [27].

Herein, we report the identification of a mutation at the stem loop of porcine pre-miR-15b and show, through *in vitro* and *in vivo* experiments, that this mutation blocks the maturation of miR-15b/16-1 and alters strand selection between miR-15b-5p and miR-15b-3p.

## Materials and methods

### Ethics statements

The protocol employed in this study was reviewed and approved by the Research Ethics Committee and the Animal Ethical Committee of the Chongqing Academy of Animal Sciences. All methods used in this study were performed in accordance with protocols approved by the Laboratory Animal Management Committee of the Chongqing Academy of Animal Sciences and the Ministry of Science and Technology of the Peoples Republic of China (Approval number: 2006398).

### Tissue and blood collection

Ear tissues were collected from Duroc (n = 298) and Landrace (n = 366) pigs residing at the 69 Pig Breeding Farm in Chongqing, China, and from Rongchang (n = 86) pigs residing at the Pig Breeding Farm of Chongqing Academy of Animal Science. The tissue specimens were used for sequencing of pri-miR-15b and SNP screening. Ear tissues and 500μl blood specimens (were collected into RNAprotect Animal Blood Tubes; Qiagen, Hilden, Germany) were collected from 6 litters of Duroc piglets (n = 60) at 0 days old residing at the 69 Pig Breeding Farm. The newborn piglets fed with breast milk of the mother sow. All efforts were made to minimize any discomfort during sample collection. All samples were not obtained from dead animals. The samples for research were obtained permission from the 69 Pig Breeding Farm and the Pig Breeding Farm of Chongqing Academy of Animal Science.

### Genotyping

Genomic DNA was extracted from the pig ear tissues by using the TIANamp Genomic DNA Kit (Tiangen, Beijing, China). PCR amplification of a 275 bp fragment containing the pre-miR-15b was carried out by using 15bF and 15bR primers (S1 Table) with reagents from the TaKaRa Taq™Polymerase Kit (TaKaRa, Dalian, China). The thermocycling reaction consisted of 35 cycles of 94°C for 30 s, 60°C for 30 s, and 72°C for 30s. The resultant PCR products were sent to GENEWIZ Biotech Co. Ltd (Suzhou, China) for sequencing.

### Plasmid construction

The 559 bp fragment containing both pri-miR-15b and miR-16-1 from either wild-type or mutant-type pigs (amplified by the respective F and R primers; S1 Table) and harboring *Xho*I and *Bam*HI restriction enzyme cleavage sites was ligated into the pmR-mCherry vector (Clontech, Mountain View, CA, USA) to generate the respective PmR-miR-15b expression vectors. The vectors were confirmed by sequencing (GENEWIZ Biotech Co. Ltd).

### Cell culture and transfection

The HEK293 cell line was purchased from the China Infrastructure of Cell Line Resources. The cells were maintained in Dulbecco’s modified Eagle’s medium supplemented with 10% fetal bovine serum and 2 mM penicillin/streptomycin. Transfections were performed in duplicate in 24-well plates to achieve a quintuplicate format and the procedure was carried out following the manufacturer’s instructions for the TransIT-2020 Transfection Reagent (Mirus Bio LLC, Madison, WI, USA).

### RNA extraction and quantitative real-time PCR (qPCR)

Total RNA was isolated from cultured cells by treatment with the Trizol Reagent (Invitrogen, Carlsbad, CA, USA) and from blood specimens of wild-type (n = 9) and mutant-type (n = 9) piglets by use of the RNeasy Protect Animal Blood Kit (Qiagen). The isolated RNA samples were reverse-transcribed into cDNA with the miScript II RT Kit (Qiagen). The samples were subjected to qPCR in the 7900HT Standard Real-Time PCR System (Applied Biosystems Inc., Hercules, CA, USA) and using gene-specific primers and reagents from the miScript SYBR Green PCR Kit (Qiagen). Mature miR-15b-5p, miR-15b-3p and miR-16 were detected by the forward primers miR-15b-5pF, miR-15b-3pF and miR-16F, respectively (S1 Table), with the MiScript Universal Primer (of the miScript II RT Kit) used as the reverse primer for all. Pri-miR-15b-F1 and pre-miR-15b-R (S1 Table) were used for detection of pri-miR-15b. Pre-miR-15b-F2 (S1 Table) and precursor-miR-15b-R were used to detect the total expression level of pri-miR-15b and pre-miR-15b. The quantified expression levels of the various miRNAs and the miR-15b precursors were normalized according to the level of mCherry gene detected by qPCR as red fluorescent protein expressed from the pmR-mCherry vector. Relative abundance of detected transcripts was calculated using the 2^−ΔΔCt^ method.

### Statistical analysis

The significance of differences among the quantitative PCR results was evaluated by Student’s t-test, with the threshold of significance set at *P* <0.01. All analyses were performed with Microsoft Excel.

## Results

### Rs334680106 is predicted to alter the thermodynamic stability and secondary structure of pre-miR-15b

**Fig 1:**
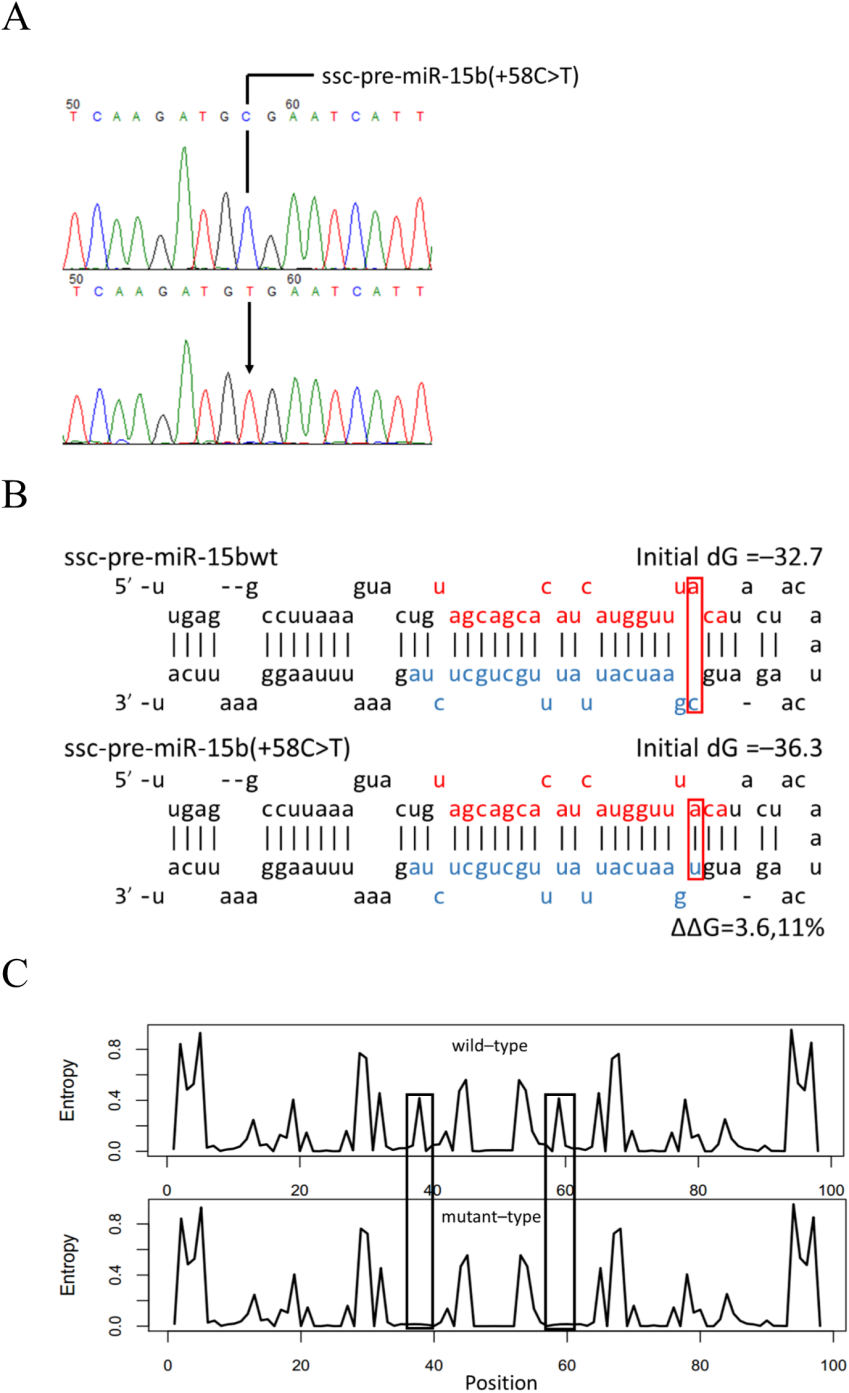
A mutation in pre-miR-15b alters its free energy. (A) A C-to-T mutation was detected in the genomic sequence of pre-miR-15b. The arrow points to the nucleotide that is mutated. (B) Secondary structures of the wild-type and mutant pre-miR-15b molecules as predicted by Mfold. The miR-15b-5p mature sequence is highlighted in red and the miR-15b-5p in blue. The mutation is indicated by a red square. Free energies calculated by Mfold are indicated.(C) The entropy of the thirty-ninth nucleotide and fifty-eighth nucleotide of the wild-type and mutant pre-miR-15b calculated by RNAfold. The thirty-ninth nucleotide and fifty-eighth nucleotide of pre-miR-15b is indicated by squares.

A 275 bp fragment of the miR-15b gene locus was amplified from genomic DNA of the Duroc (n = 298) and Landrace (n = 366) pig breeds, both originated from Europe, and of the Rongchang pig breed (n = 86), a Chinese native. Sequencing revealed a C-to-T variation (+58C>T) at position 58 in the miR-15b precursor (pre-miR-15b) (Fig 1A), which had been previously reported in the dbSNP database (rs334680106). Genotyping revealed that the variation was present only in the Duroc and Landrace breeds, with frequencies of the non-reference allele (T) being 27.0% and 3.0%, respectively (Table 1).

**Table 1:** Allele and genotype frequencies of pre-miR-15b in the three different pig breeds.

		Frequencies		
		Landrace	Duroc	Rongchang
Genotype	CC	0.94(344)	0.55(164)	1(86)
	CT	0.06(22)	0.36(107)	0(0)
	TT	0	0.09(27)	0(0)
Allele	C	0.97	0.73	1
	T	0.03	0.27	0

Numbers in brackets are population size tested that belong to the respective genotypes.

The RNA structure prediction tools of Mfold [28] and RNAfold (http://nhjy.hzau.edu.cn/kech/swxxx/jakj/dianzi/Bioinf4/miRNA/miRNA1.htm) were then used to simulate the secondary structures and free energies of the wild-type and mutant-type hairpin-shaped pre-miR-15b. The rs334680106 mutation was predicted to generate a C-to-U mutation at the first nucleotide of the 5′ end of miR-15b-3p, creating a U-A base pair in place of the C-A mismatch (Fig 1B). The mutant-type pre-miR-15b product was then predicted to have a free energy that was 11.01% lower than that of the wild-type product (Fig 1B). The mutant pre-miR-15b product was also predicted to have substantially decreased entropy for the thirty-ninth nucleotide at the 3′ end of the miR-15b-5p strand and for the fifty-eighth nucleotide at the 5′ end of the miR-15b-3p strand (Fig 1C). Thus, the rs334680106 mutation is likely to alter the thermodynamic stability and the secondary structure of pre-miR-15b, both features which may affected the biogenesis of miR-15b.

### Rs334680106 alters miRNA expression *in vitro*

**Fig 2:**
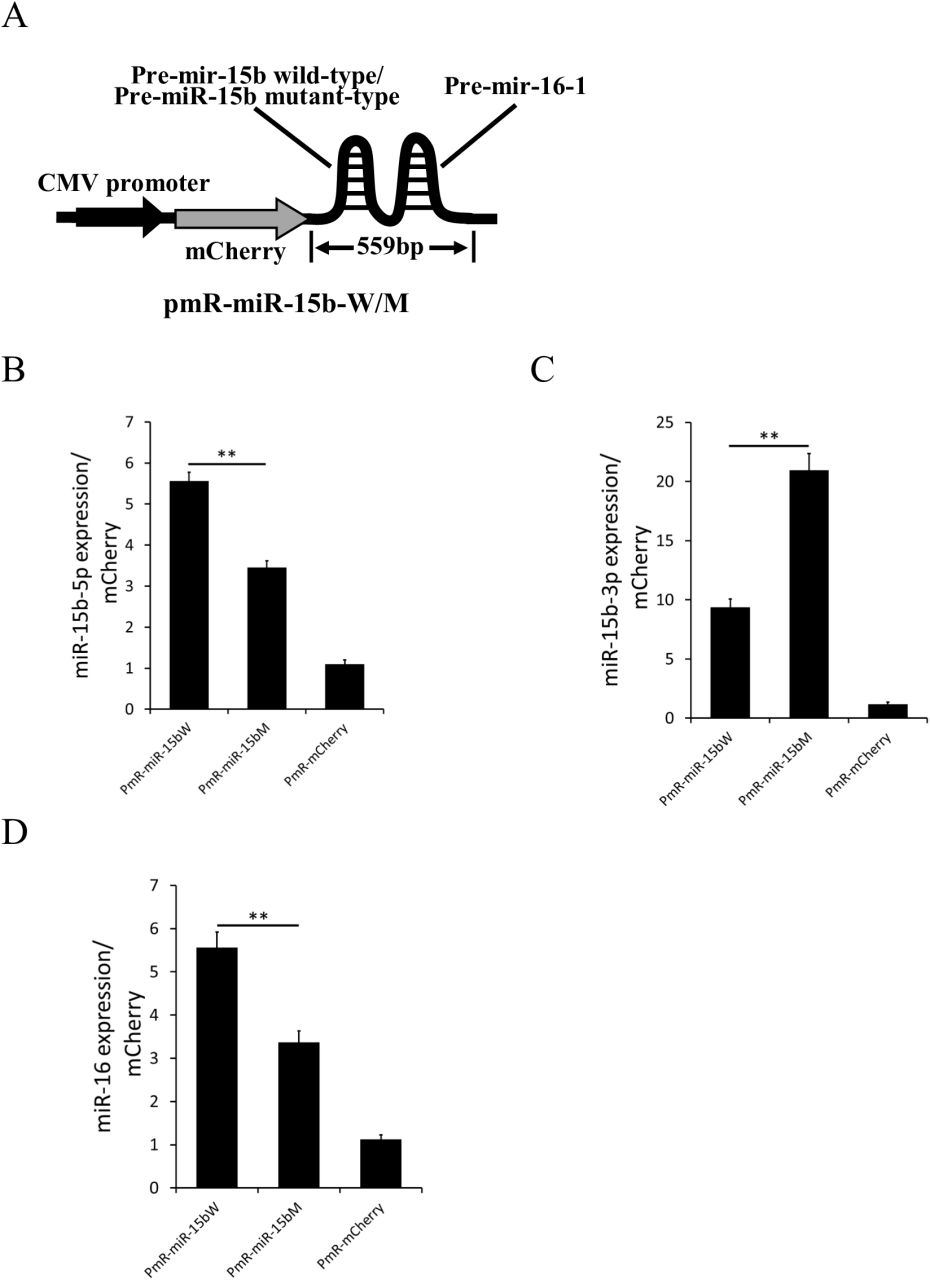
Rs334680106 alters miRNA expression *in vitro*. (A) Schematic representation of PmR-miR-15b-wild-type (W) and PmR-miR-15b-mutant (M) overexpression vectors. Quantitative RT–PCR was used to measure level of mature miR-15b-5p(B), miR-15b-3p(C) and miR-16(D) in 293 cells transduced by PmR-miR-15b-W, PmR-miR-15b-M and the control PmR-mCherry expression vector. MCherry was used as the internal control. Transfection was repeated three times. Data are presented as mean ± SD. **P<0.01.

To explore whether rs334680106 influences the biogenesis of miR-15b, wild-type and mutant-type sequences of the porcine miR-15b/miR-16-1 cluster were inserted into the mammalian express vector PmR-mCherry (to generate PmR-miR-15bW and PmR-miR-15bM vectors) and over-expressed in the HEK293 cell line (Fig 2A). The expression level of mature miRNA was verified by quantitative RT–PCR, and the expression of mCherry was used to normalize the transfection efficiency. As miR-15b-5p is the dominate strand of miR-15b [29], miR-15b-5p was detected first and the results showed that its expression was significantly down-regulated in the mutant (Fig 2B); subsequent detection of miR-15b-3p showed its expression to be significantly up-regulated in the mutant (Fig 2C). Interestingly, detection of miR-16 showed that its expression was also down-regulated in the mutant, to an extent that was similar to miR-15b-5p. These results suggested that rs334680106 impacted the efficiency of the miR-15b processing, generating opposite effects on the 5p and 3p strands of the mature miRNA. Moreover, the rs334680106 mutation in miR-15b appeared to be able to also affect the biogenesis process of miR-16-1, which is located in the same cluster as miR-15b (Fig 2D).

### Rs334680106 blocks the pri-miRNA to pre-miRNA processing of miR-15b

**Fig 3:**
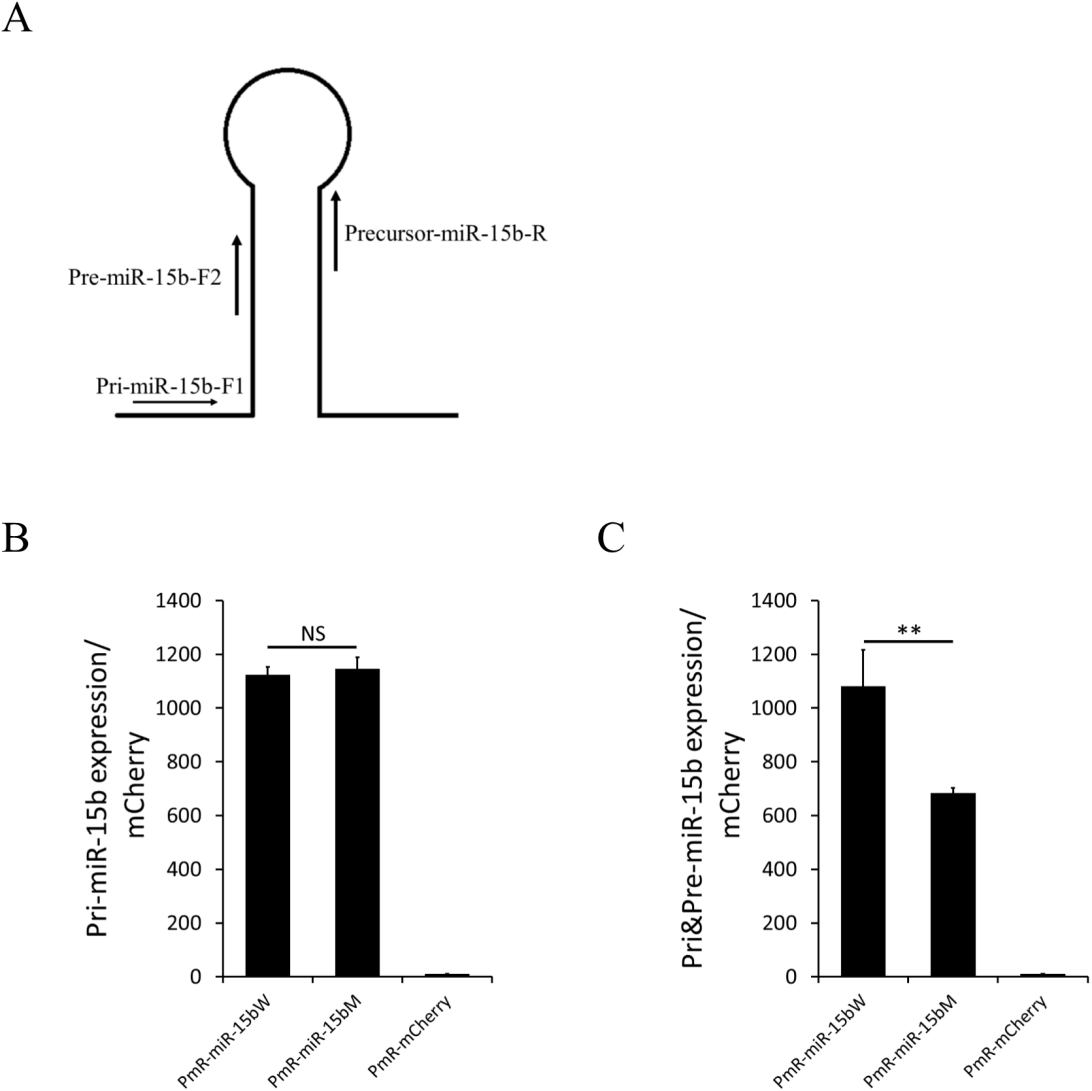
Rs334680106 blocks the processing of pri-miR-15b to pre-miR-15b. (A) Schematic diagram of the specific primers designed to amplify pri-miR-15b and pre-miR-15b; the pri-miR-15b-F1 and precursor-miR-15b-R were used to amplify pri-miR-15b, and the pri-miR-15b-F2 and precursor-miR-15b-R were used to amplify the total level of pri-miR-15b and pre-miR-15b. Quantitative RT–PCR was used to measure level of pre-miR-15b (B) and pri-miR-15b (C) in 293 cells transduced by PmR-miR-15b-W, PmR-miR-15b-M and the control PmR-mCherry expression vector. MCherry was used as the internal control. Transfection was repeated three times. Data are presented as mean ± SD. **P<0.01.

To explore which step of the biogenesis processing was affected by rs334680106, specific primers (S1 Table) were designed to identify expression of the pri-miR-15b and pre-miR-15b intermediate products [30] (Fig 3A). No difference was detected in the expression levels of pri-miR-15b for the wild-type and mutant-type (Fig 3B). However, the overall level of pri-miR-15b plus pre-miR-15b was remarkably lower for the mutant-type (57.9% compared to the wild-type) (Fig 3C). These data indicated that the rs334680106 mutation did not alter the primary precursor transcription of miR-15b but did block the pri-miRNA processing to pre-miRNA.

### Rs334680106 impacts the strand selection between miR-15b-5p and miR-15b-3p

**Fig 4:**
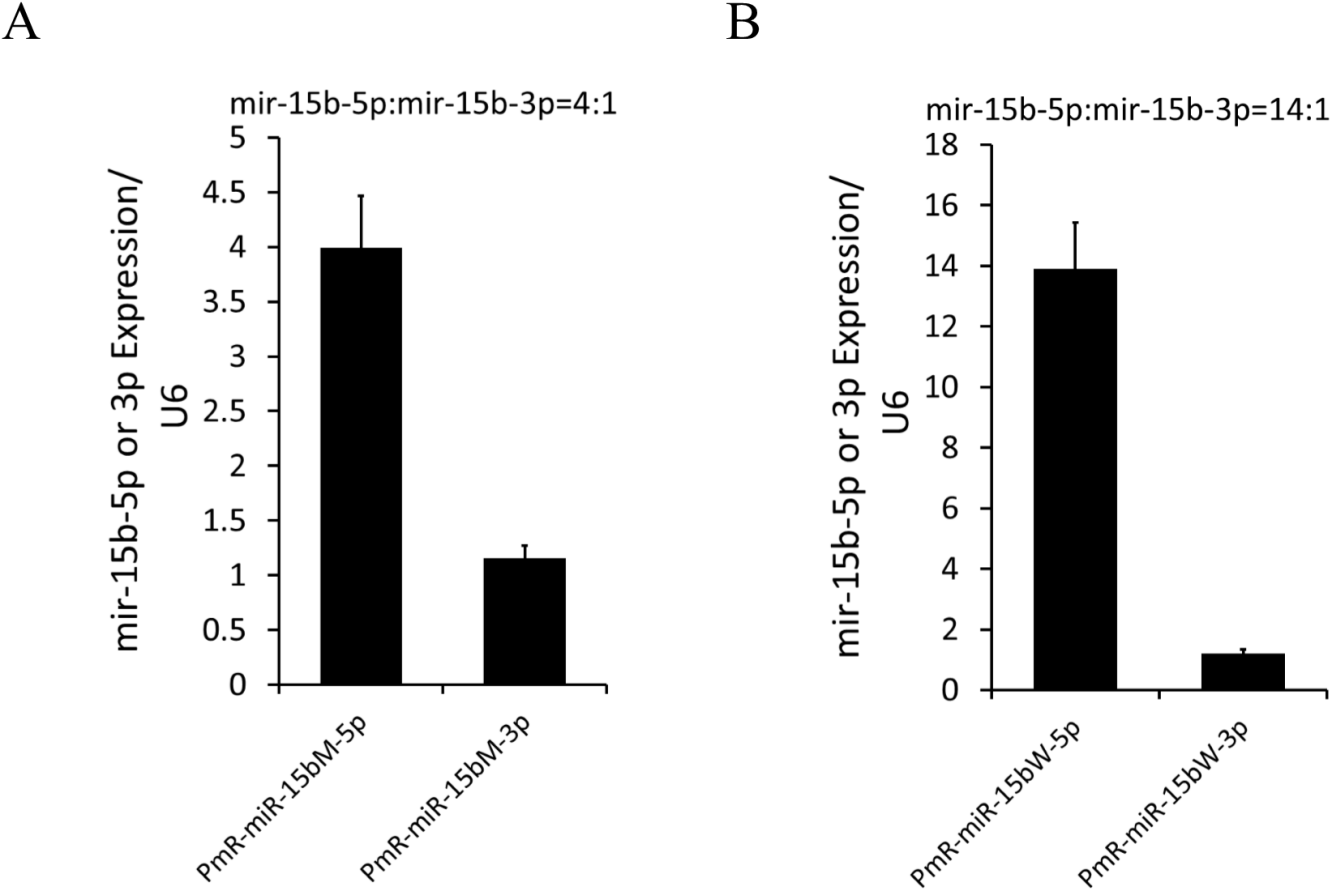
**The expression ratio of miR-15b-5p to miR-15b-3p in wild-type (A) and mutant-type (B) groups.** Quantitative RT–PCR was used to measure level of mature miR-15b-5p and miR-15b-3p in 293 cells transduced by PmR-miR-15b-W(A), PmR-miR-15b-M(B). U6 was used as the internal control. Transfection was repeated three times. Data are presented as mean ± SD.

After microprocessor processing, the pre-miRNA is cleaved by Dicer to release the ~22 nt miRNA duplex [10]. Then, the AGO protein selects one strand of the miRNA duplex as the guide strand to form the major mature miRNA, a small number of passenger strands can also form other mature miRNAs (designated here as miRNA*); this feature is evidenced by a strong bias towards the guide strand in the mature miRNA pool [31]. Considering the opposite-direction effects of rs334680106 on miR-15b-5p and miR-15b-3p cited above, we next aimed to determine whether rs334680106 could affect the strand selection between miR-15b-5p and miR-15b-3p by evaluating the expression ratio of the two strands in our experimental system. The ratio was found to be significantly different between the mutant-type and the wild-type (Fig 4A and 4B), indicating that rs334680106 impacts the strand selection step for the composition of RISC.

### Rs334680106 alters miRNA expression *in vivo*

**Fig 5:**
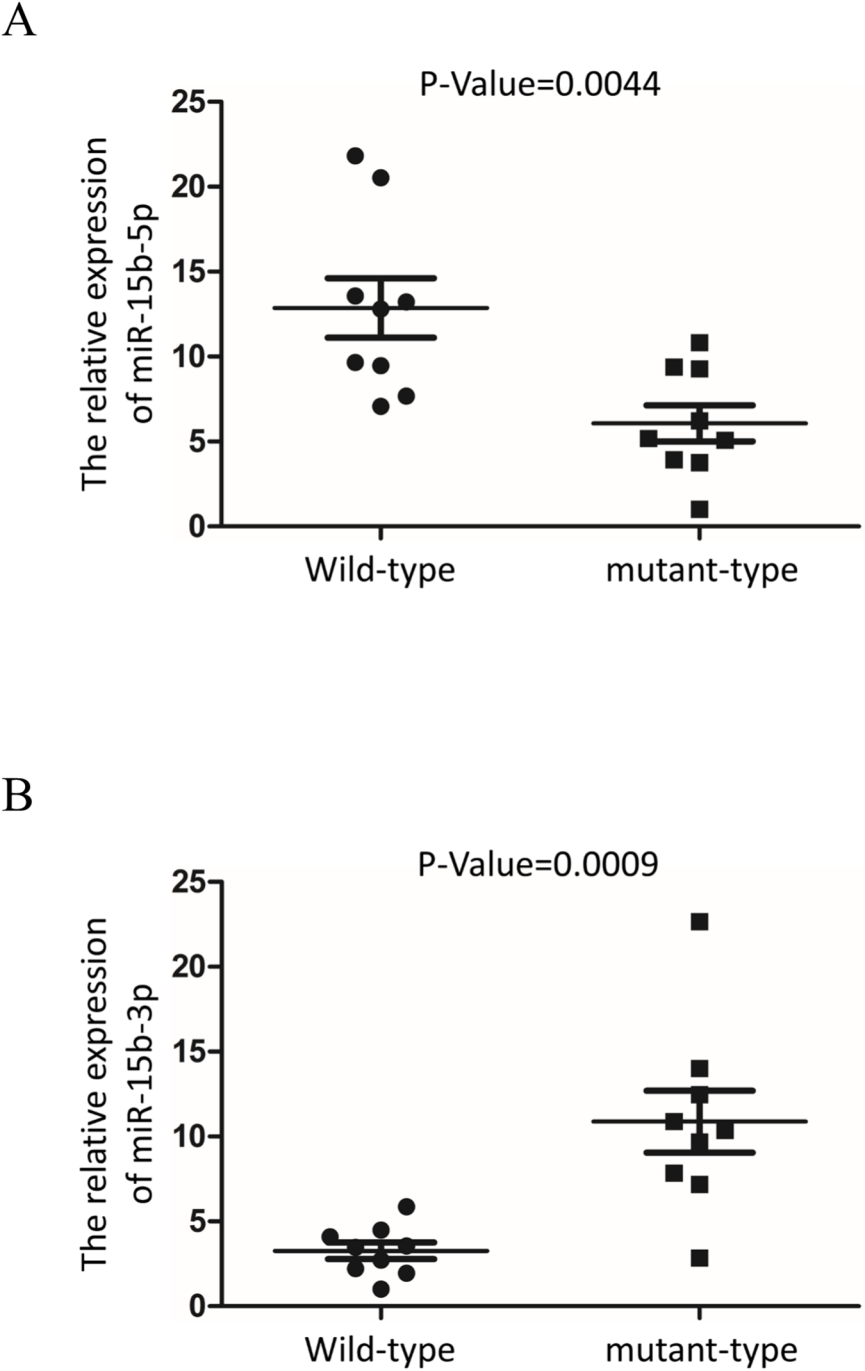
Rs334680106 alters miRNA expression *in vivo*. Expression level of miR-15b-5p (A) and miR-15b-3p (B) in blood of the wild-type (n = 9) and mutant-type (n = 9) piglets. U6 was used as the internal control. *P*-value are indicated.

To further investigate whether rs334680106 effects miR-15b expression *in vivo*, blood specimens from wild-type (n = 9) and mutant-type (n = 9) newborn piglets were examined and the latter was found to have significantly lower expression of miR-15b-5p and significantly higher expression of miR-15b-3p (Fig 5). These findings were concordant with the results of the *in vitro* experiments cited above.

## Discussion

MiRNAs are involved in nearly all cellular pathways, contributing to molecular regulation of normal physiological processes, ranging from development to cell cycling, and their dysregulation has been impacted in an equally diverse range of pathological processes, ranging from oncogenesis to autoimmune diseases [2,32,33]. The mechanisms that control miRNA biogenesis are precisely regulated, from both spatial and temporal perspectives; such dynamic regulation is necessary to control the myriad targeting of miRNAs to the multitudes of genes that they act upon to finely modulate expression [31].

Variations in miRNA sequences can cause their functional dysregulation [16,20,34]. In the present study, we found that rs334680106 not only blocks the processing of pri-miRNA to pre-miRNA and alters the expression level of miR-15b but also influences the strand selection of miR-15b; furthermore, the significant genetic effect of rs334680106 was confirmed in blood specimens taken from living animals. We were surprised to find that the rs334680106 mutation also influenced the biogenesis of miR-16-1, which is located in the genomic cluster with miR-15b. To our knowledge, this is the first reported evidence of a single mutation in pre-miRNA that produces multiple effects among various steps in miRNA biogenesis. Collectively, these data imply that mutations in miRNA precursors could have complex effects on miRNA biogenesis and have potential for profound genetic effects.

Several studies have demonstrated that mutations in miRNA genes can block the microprocessor-mediated step of biogenesis, creating variability in phenotype, and are associated with disease pathogenesis [14,35]. Our data provide evidence that a mutation in the pri-miRNA can disrupt the ability of that pri-miRNA to be processed into a pre-miRNA. The observation of the fold-change in expression level of the miR-15b-5p of the mutant-type, compared to that of the wild-type, being approximately equal to that of pre-miR-15b indicated that the mutant-associated reduction in expression was caused by blockade at a step involving the microprocessor or earlier.

The Drosha ribonuclease III component of the microprocessor uses single-stranded (ss)RNA and double-stranded (ds)RNA structural elements of the pri-miRNA for distinguishment and subsequent cutting [36,37]. DGCR8, the other component of the microprocessor, interacts with its particular dsRNA-binding domains in the stem sequence of pri-miRNA, with the result of enhancing the microprocessor-processing efficiency [37,38]. Although the rs334680106 mutation was not predicted to alter the overall ssRNA-dsRNA structure that is otherwise essential for the Drosha-mediated processing reaction, it does introduce a U-A base-pairing in the pre-miR-15b, with the result of decreasing the conformation of the RNA bulge in the stem of the predicted precursor structure. Therefore, we theorize that this mutation may block the microprocessor-processing by affecting the affinity of DGCR8, and not that of Drosha.

Strand selection is a biased procedure in AGO-mediated miRNA biogenesis, manifesting as expression disequilibrium between mature miRNA and its partner miRNA* [11,12]. There are two rules in strand selection, regarding thermodynamic stability of the two ends of the miRNA/miRNA* duplex and the strand composition respectively, and these were experimentally validated in the present study. In particular, the observed selection bias favored the strand with 1) the relatively lower thermodynamic stability at the 5′ terminus [11,12] and 2) having a U at the first nucleotide position of the 5′ end [39–41], as the guide strand. The rs334680106 mutation was found to differentially affect the expression levels of the miR-15b-5p and miR-15b-3p strands, reducing the former and increasing the latter. Ultimately, this manifested a different expression ratio for the two miR-15b strands, as compared to that of wild-type miR-15b, and suggested that rs334680106 influences the strand selection between miR-15b-5p and miR-15b-3p. The rs334680106 mutation created a U-A base pair in place of the C-A mismatch, introducing a G-U bulge in the miRNA/miRNA* duplex at the 5′ end of miR-15b-3p and making it more unstable than the wild-type; furthermore, the first nucleotide of miR-15b-3p become a U in the mutant-type, enhancing the potential of the AGO protein to select more miR-15b-3p for formation of RISC. Hence, considering these features in the context of the two rules of strand selection (described above), we theorize that the thermodynamic instability and the presence of the U at the 5′ end of miR-15b-3p caused the increased expression of miR-15b-3p that was observed.

It is important to note that although the rs334680106 mutation influenced strand selection between miR-15b-5p and mir-15b-3p and increased expression of miR-15b-3p, the main expression of the mature miR-15b product remained as mir-15b-5p, similar to that for the wild-type. Almost certainly, then, there are other important rules for strand choice underlying this process. In a previous study, Sun et al. had identified a T/G transversion at the first nucleotide of the miR-934-5p strand that altered miRISC strand loading bias by increasing 5′ end thermodynamic stability of miR-934-3p and reducing that of miR-934-5p, resulting in increased expression of 3p and decreased expression of 5p [19].

Thus, the present study represents the second instance in which a sequence variation in a pre-miRNA is shown to affect strand selection between miRNA and miRNA*. Passenger strand-produced miRNA* can also exert different function(s) than the guide strand and may represent an important regulatory features that is yet to be fully recognized [42–44]. Further investigations should aim to determine whether and how the rs334680106 mutation affects the function of miR-15b-3p and to identify other mutations in miRNA genes that can alter strand selection.

It is almost certain that the regulatory process of miRNA biogenesis is more complex *in vivo* than in an experimental *in vitro* system. It is common for results that are obtained in a cultured/immortalized cell line to not be repeated using an experimental *in vivo* system or under the real-life physiological conditions of living animals. However, our results from cultured cells, showing significant effects of rs334680106 on miR-15b expression, were similar to those of living pigs naturally harboring the mutation. Thus, we feel confident in pursuing future investigations into the influence of rs334680106 on the biogenesis of miR-15b.

In the genome, some miRNAs are located in proximity to one another, constituting a polycistronic transcription unit [45]. MiRNAs in the same cluster are generally co-transcribed, but the individual miRNAs can also be regulated at the post-transcriptional level [31]. Interestingly, our results show that a SNP in miR-15b can affect the biogenesis process of another miRNA, namely miR-16-1 which is located in the same cluster as miR-15b, and that the mutation-related fold-change in expression of miR-16 is approximately equal to that of miR-15b-5p. It is possible, even likely, that rs334680106 might block affinity of the microprocessor to the primary transcript that includes both miR-15b and miR-16-2. To our knowledge, this is the first report of a single mutation in one miRNA capable of influencing another miRNA located in the same cluster. We propose, based on our findings, that SNPs are not only able to disrupt the cleavage of proximal miRNA but also that of distal miRNAs; however, more focused investigations are needed to understand the extent and mechanism of these kinds of effects.

In summary, the rs334680106 mutation in pre-miR-15b alters biogenesis of the miR-15b\16–1 cluster and the strand selection of miR-15b. Further exploration of the relationship between this +58 C>T mutation and the function of miR-15b may provide intriguing results generalizable to the overall field of miRNA regulation of physiologic and pathologic processes. Regardless, our study revealed the importance of miRNA mutations on their expression, and subsequent availability for functional contributions, through their involvement in miRNA biogenesis.

## Supporting information

S1 Table Information of primers used. (DOCX)

S2 Table The ARRIVE guidelines checklist. (DOCX)
