# Supplementary material for: A mutation in porcine pre-miR-15b alters the biogenesis of MiR-15b\16-1 cluster and strand selection of MiR-15b
Source: PLoS One. 2017 May 24;12(5):e0178045. doi: 10.1371/journal.pone.0178045 (PMC5443575; doi:10.1371/journal.pone.0178045)
Supplement: S1 Table — (DOCX) [file pone.0178045.s001.docx]

**Supporting Information**

**S1 Table. Information of Primers used**

| Name | Sequence(5′-3′) | Annealing Temperature  (°C) | Application |
| --- | --- | --- | --- |
| 15bF | GGTTCTCTCGTCCTTGTTTTTG | 60 | genotyping |
| 15bR | AGAGCGGAACAAGTATGTCAGT |  |  |
| F | CTCGAGCACATAAGTGGGAAGAAGAAGACA | 60 | Plasmid construction |
| R | GCTCGAATTCCAGAGGTCTCAGGGCTATGC |  |  |
| miR-15b-5pF | TAGCAGCACATCATGGTTTACA | 60 | Expression of miR15b-5p |
| miR-15b-3pF | CGAAUCAUUAUUUGCUGCUCUA | 60 | Expression of miR15b-3p |
| miR-16F | TAGCAGCACGTAAATATTGGCG | 60 | Expression of miR16 |
| Pri-miR-15b-F1 | GGAGTTTTTCCCTTTTGGATG | 60 | quantitation of pri- and pre-miR-15b |
| pre-miR-15b-R | ATAATGATTCGCATCTTGATTGTAG |  |  |
| Pre-miR-15b-F2 | GGAGTTTTTCCCTTTTGGATG |  |  |
| mCherryF | GTGACCGTGACCCAGGACT | 60 | quantitation of red fluorescent protein gene |
| mCherryR | GTCGGAGGGGAAGTTGGT |  |  |
| U6F | CTCGCTTCGGCAGCACA | 60 | Relative quantification of miR15b-5p to miR15b -3p |
| U6R | AACGCTTCACGAATTTGCGT |  |  |
