# Supplementary material for: A mutation in porcine pre-miR-15b alters the biogenesis of MiR-15b\16-1 cluster and strand selection of MiR-15b
Source: PLoS One. 2017 May 24;12(5):e0178045. doi: 10.1371/journal.pone.0178045 (PMC5443575; doi:10.1371/journal.pone.0178045)

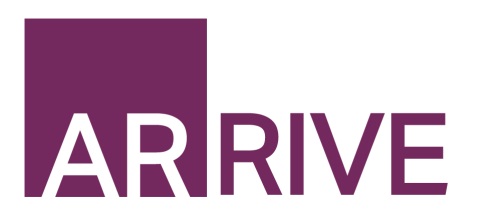


S2 Table. The ARRIVE Guidelines Checklist

Animal Research: Reporting In Vivo Experiments

Carol Kilkenny^1^, William J Browne^2^, Innes C Cuthill^3^, Michael Emerson^4^ and Douglas G Altman^5^

*^1^The National Centre for the Replacement, Refinement and Reduction of Animals in Research, London, UK, ^2^School of Veterinary Science, University of Bristol, Bristol, UK, ^3^School of Biological Sciences, University of Bristol, Bristol, UK, ^4^National Heart and Lung Institute, Imperial College London, UK, ^5^Centre for Statistics in Medicine, University of Oxford, Oxford, UK.*

|  | | ITEM | RECOMMENDATION | Section/ Paragraph |
| --- | --- | --- | --- | --- |
| 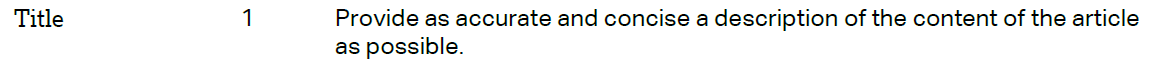 | | | Results,  Paragraph 6 |  |
| 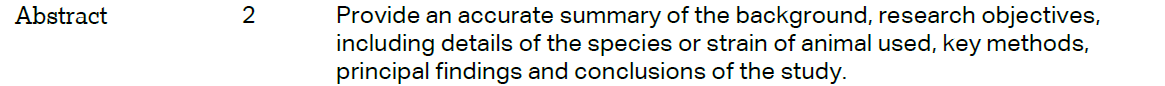 | | | Results,  Paragraph 6 |  |
| INTRODUCTION | | |  |  |
| 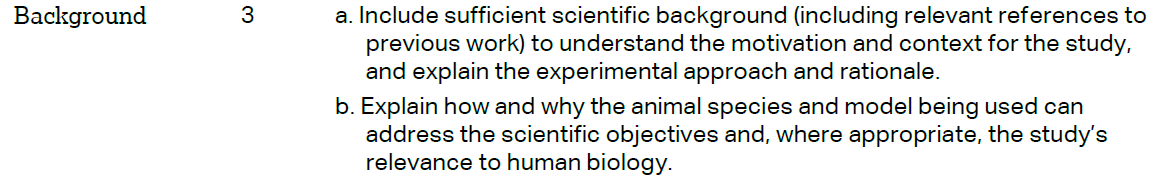 | | | Results,  Paragraph 6 |  |
| 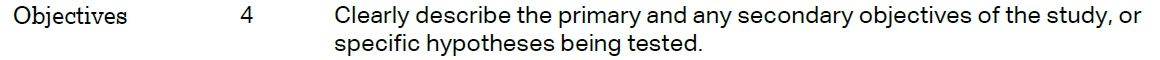 | | | Results,  Paragraph 6 |  |
| METHODS | | |  |  |
| 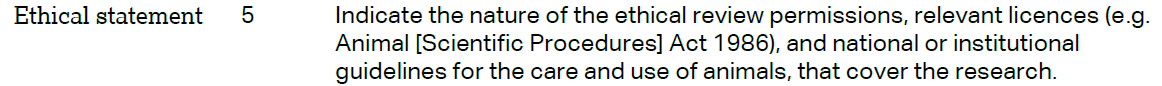 | | | Materials and methods,  Paragraph 1 and 2 |  |
| 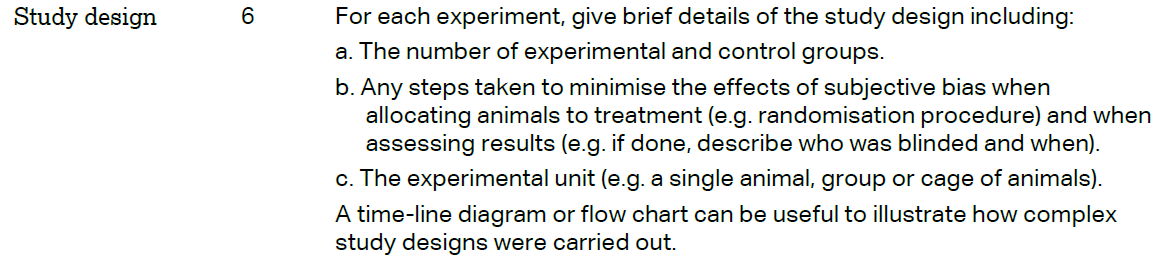 | | | Results,  Paragraph 6 |  |
| 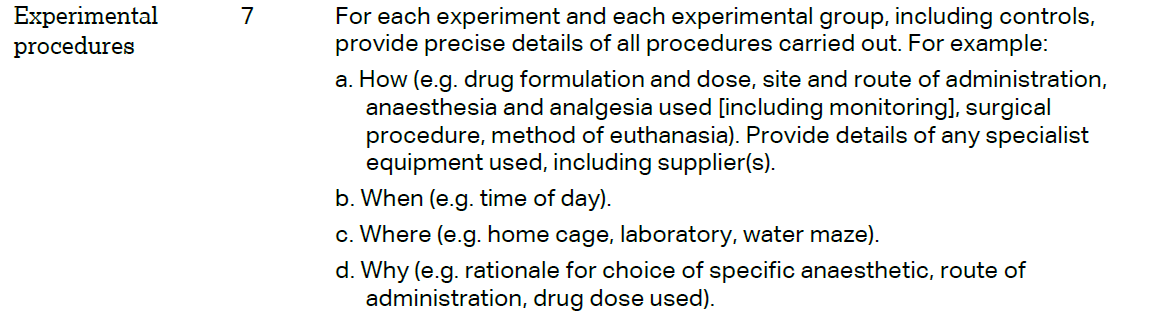 | | | Materials and methods,  Paragraph 2 |  |
| 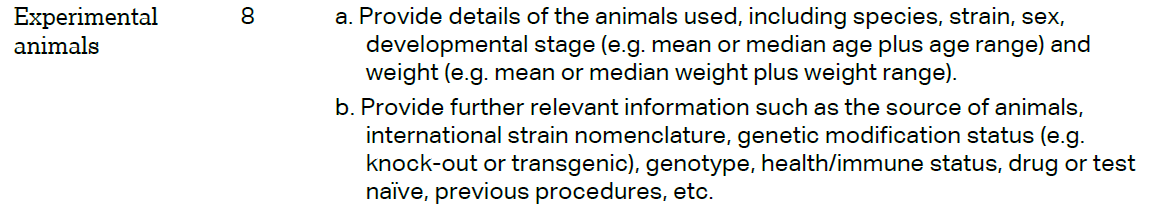 | | | Materials and methods,  Paragraph 2 |  |

The ARRIVE guidelines. Originally published in *PLoS Biology*, June 2010^1^

| 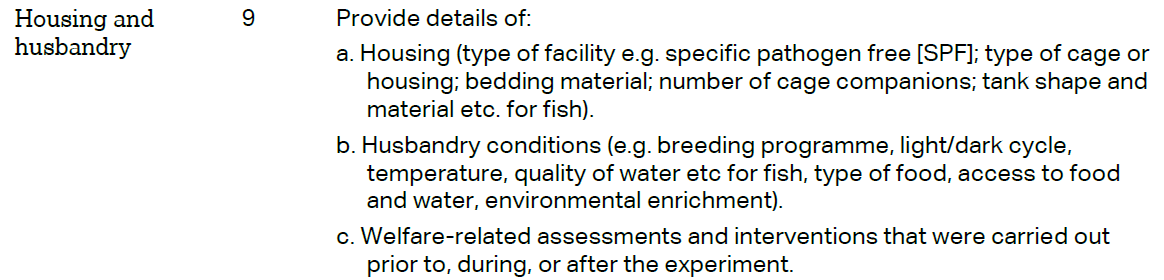 | Materials and methods,  Paragraph 2 | |
| --- | --- | --- |
| 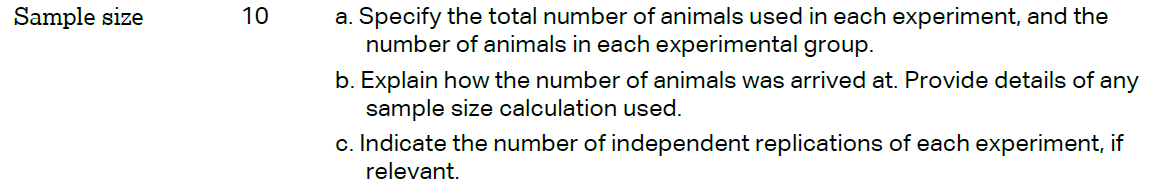 | Results,  Paragraph 6 | |
| 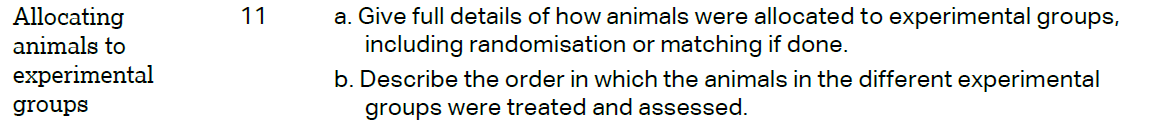 | Results,  Paragraph 6 | |
| 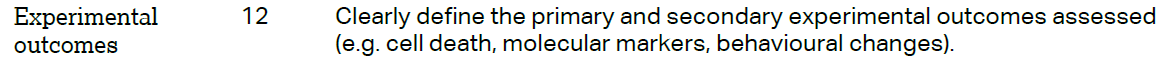 | Results,  Paragraph 6 | |
| 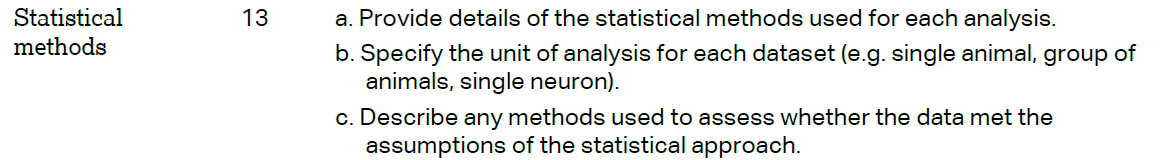 | Materials and methods,  Paragraph 6 and 7 | |
| RESULTS |  | |
| 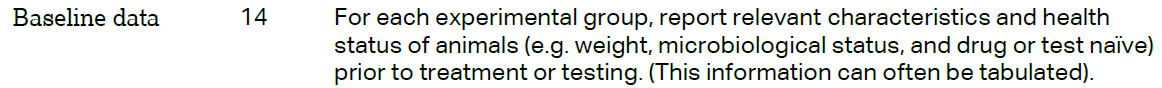 | None | |
| 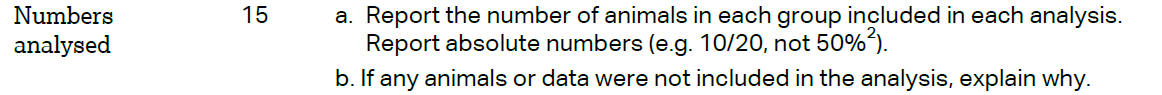 | Results,  Paragraph 6 | |
| 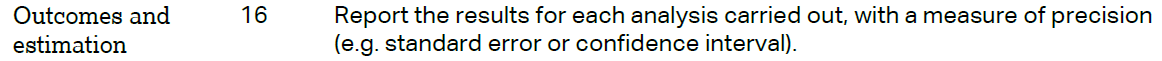 | Results,  Fig.5 | |
| 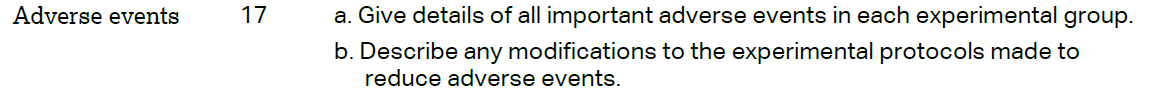 | None | |
| DISCUSSION |  | |
| 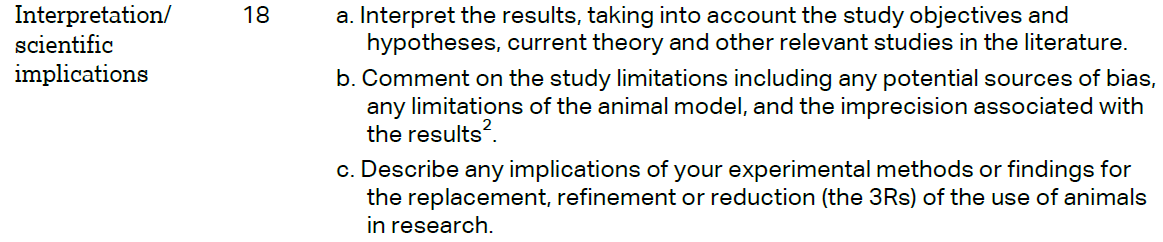 | None | |
| 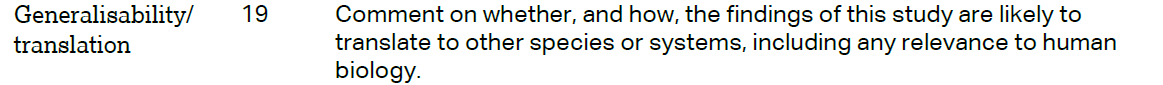 | None | |
| 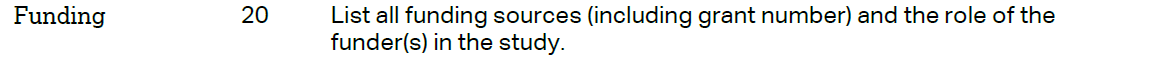 | | In the information of Submission |


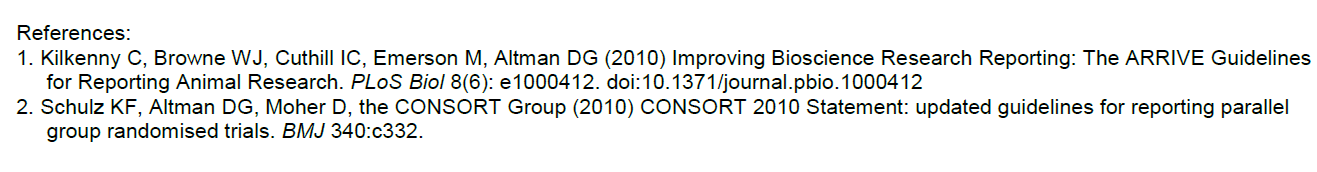

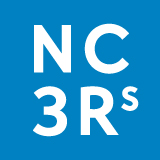

Supplement: S2 Table — (DOCX) [file pone.0178045.s002.docx]
